# ﻿*Telipogon rojasiae* (Orchidaceae, Oncidiinae), a new species from relict forests on the western slopes of the northern Peruvian Andes

**DOI:** 10.3897/phytokeys.265.164394

**Published:** 2025-10-16

**Authors:** Carlos Martel, Alex G. Diaz Hernández, Gabriel A. Iturralde, Benjamín Collantes

**Affiliations:** 1 Royal Botanic Gardens, Kew, TW9 3AB, Richmond, UK Royal Botanic Gardens, Kew Richmond United Kingdom; 2 Instituto de Ciencias Ómicas y Biotecnología Aplicada, Pontificia Universidad Católica del Perú, 15088, Lima, Peru Pontificia Universidad Católica del Perú Lima Peru; 3 Herbario Pedro Ruiz Gallo, Facultad de Ciencias Biológicas, Universidad Nacional Pedro Ruíz Gallo, 1400, Lambayeque, Peru Universidad Nacional Pedro Ruíz Gallo Lambayeque Peru; 4 Grupo de Investigación en Biodiversidad, Medio Ambiente y Salud (BIOMAS), Carrera de Ingeniería en Agroindustria, Facultad de Ingenierías y Ciencias Aplicadas, Universidad de Las Américas, UDLA, Vía a Nayón, Quito 170124, Ecuador Universidad de Las Américas Quito Ecuador; 5 Inka Terra Association, 15074, Lima, Peru Inka Terra Association Lima Peru

**Keywords:** Ecuador, endemic species, Oncidiinae, *Telipogon ecuadorensis*, *Telipogon frymirei*, *Telipogon montufarianus*, Ecuador, especie endémica, Perú, *Telipogon ecuadorensis*, *Telipogon frymirei*, *Telipogon montufarianus*

## Abstract

*Telipogon rojasiae*, from the western Andean slopes of northern Peru, is described as a new species. *Telipogon rojasiae* is similar to *Telipogon montufarianus*, which is only known from southern Ecuador, but differs in its cream-yellow flowers heavily stained with red-vinaceous (vs. bright yellow flowers), the sub-rhombic to obovate petals (vs. elliptic petals), the number of veins in the petals (9–10 veins vs. 5 veins) and labellum (26 veins vs. 16–19 veins), and its sagittate callus (vs. widely subcordiform callus). We provide a description, illustrations, photographs, and information about the habitat of this new species. Furthermore, we discuss the identity and occurrence of morphologically similar species. The discovery of this new entity highlights the importance of preserving the relict forests of the western Andean slopes in north-west Peru and south-west Ecuador.

## Abstract (es)

*Telipogon rojasiae*, de los Andes occidentales del norte de Perú, es descrita como una nueva especie. *Telipogon rojasiae* es similar a *Telipogon montufarianus*, la cual solo se conoce de Ecuador, pero se diferencia por las flores cremas amarillas tenidas de rojo vino (vs. flores amarillo brillantes), los pétalos sub-rómbicos a obovados (vs. pétalos elípticos), el número de venas en los pétalos (9–10 venas vs. 5 venas) y el labelo (26 venas vs. 16–19 venas), y el callo sagitado (vs. el callo ampliamente sub-cordiforme). Proporcionamos una descripción, ilustraciones, imágenes e información sobre el hábitat de esta nueva especie. Además, analizamos la identidad y la presencia de la nueva entidad y de especies morfológicamente similares. El descubrimiento de la nueva entidad resalta la importancia de preservar los bosques relictos de la vertiente occidental de los Andes en el norte del Perú.

## ﻿Introduction

*Telipogon* Kunth is a Neotropical orchid genus distributed from southern Mexico and the Caribbean to Bolivia (Martel and Nauray Huari 2013). *Telipogon* plants grow at mid and high elevations (500–3800 m), although most species are found in mountain forests between 2200 and 3500 m. In its current circumscription, *Telipogon* includes 258 species (POWO 2025). However, many more remain undescribed, since *Telipogon* species often have highly restricted distributions (Nauray and Galán 2008; Martel 2016), and new explorations continue to reveal new taxa, with several proposed each year (e.g. Pupulin 2022; Iturralde et al. 2023; Jiménez et al. 2024). Most of this richness is found in the Andean cloud forests of Colombia, Ecuador, and Peru, where around 70% of the known *Telipogon* species occur (POWO 2025).

In Peru, *Telipogon* is currently represented by more than 50 species (Martel et al. 2020), most of which occur between 2000 and 3500 m on the eastern slopes of the Andes, characterised by year-round humidity and predominantly forested environments. Conversely, the western slopes of the Andes are dominated by the Andean shrubland ecosystem (Matorral Andino in Spanish; Ministerio del Ambiente 2019), which is characterised by a dry climate and predominance of sparse shrubland along with grassland. However, due to the Huancabamba depression – an east–west depression through the Andes between southern Ecuador and northern Peru – humid air from the east reaches some areas of the western slopes above 8° latitude south. This has made possible the occurrence of other types of ecosystems, such as the relict montane forests (“bosque relicto montano de vertiente occidental” in Spanish; Ministerio del Ambiente 2019), which are characterised by sparse humid forests. These forest remnants are biodiversity-rich ecosystems, which are unfortunately disappearing rapidly due to the expansion of human activities (Weigend et al. 2005b). Efforts to better characterise the plant diversity of this ecosystem type in Peru have taken place over the last few decades (Weigend et al. 2005a), and orchids have gained more attention during the last 10 years (e.g. Chamaya Gonzáles 2023).

As part of a survey of orchid diversity in the relict montane forests of northern Peru, one of the authors (AGDH) recorded and collected material of an unknown *Telipogon* species. After careful revision, we concluded that the material belonged to a previously undescribed species. Therefore, we describe and illustrate it here.

## ﻿Materials and methods

Specimens of the new species were found during botanical expeditions on the western slopes of the Peruvian Andes, in the departments of Lambayeque and Cajamarca, in 2016 and 2023. Flowers were collected and preserved in spirit, whereas vegetative parts were pressed and dried and later used to prepare the line drawing and composite plate. In situ photographs were taken using a Nikon D5000 camera fitted with macro lenses of 18–55 mm and 105 mm. The collected specimens were compared with those deposited in Peruvian and Ecuadorian herbaria, as well as with descriptions and photographs from relevant literature (e.g. Dodson 1984, 2002; Medina et al. 2019). The specimens were subsequently deposited at the herbarium of the Universidad Nacional Pedro Ruiz Gallo (PRG). A preliminary conservation assessment was prepared following IUCN (2024) criteria, based on area of occupancy and extent of occurrence data, using the GeoCAT tool (Bachman et al. 2011). Descriptions of morphological characters followed the botanical terminology established by Beentje (2012).

## ﻿Results

### ﻿Taxonomic treatment

#### ﻿ Telipogon rojasiae

Taxon classificationPlantaeAsparagalesOrchidaceae

C.Martel, A.Diaz & Iturralde sp. nov.

76BB6CB2-35B1-5DDB-842D-13C67C3AD11E

urn:lsid:ipni.org:names:77370794-1

Figs 1, 2, 3

##### Type.

Peru • Lambayeque. Prov. Ferreñafe, [Dist. Incahuasi,] Tungula, [geographical coordinates omitted for conservation purposes], 3369 m, 29 Jan 2023, *Alex G. Diaz H. 493* (***holotype***: PRG! [Accession N° 20392]. ***isotype***: PRG! [Accession N° 20392]).

##### Diagnosis.

*Telipogon rojasiae* is most similar to *Telipogon montufarianus* but differs by its cream-yellow flowers heavily stained with red vinaceous (vs. bright yellow flowers), the sub-rhombic to obovate petals, 15–17 × 11–12 mm (vs. elliptic petals, 12 × 8 mm), the greater number of veins in the petals (9–10 veins vs. 5 veins) and labellum (26 veins vs. 16–19 veins), and its sagittate callus (vs. a widely sub-cordiform callus).

**Figure 1.**
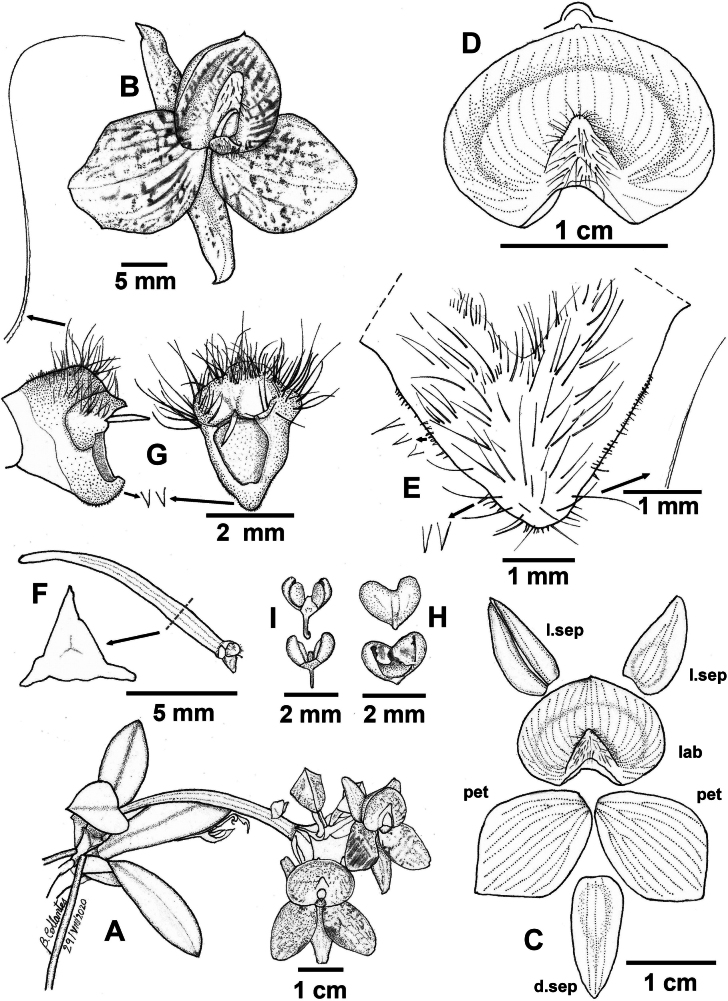
*Telipogon rojasiae* A. Habit; B. Flower, ¾ view; C. Dissected flower with expanded perianth (d.sep: dorsal sepal; l.sep: lateral sepal; pet: petal; lab: labellum), all in frontal view except one lateral sepal; D. Expanded labellum, frontal view with details of the apex; E. Callus, frontal view with details of the setae; F. Pedicel, ovary and column, lateral view with details of the ovary cross-section; G. Column, frontal and lateral views with details of the setae; H. Anther cap, frontal and ventral views; I. Pollinarium, frontal and ventral views.

##### Description.

Epiphytic ***herb***, erect, sympodial, acaulous in young plants and with a visible short stem in older plants, up to 15 cm including the inflorescence. ***Roots*** terete, 1.6–2.2 mm in diameter. ***Stem*** abbreviated, forming pseudobulb-like structures on mature plants. ***Leaves*** 2.5–5.9 × 0.6–1.1 cm, 3–9, subcoriaceous, lanceolate-oblong to narrowly sub-elliptic, entire, acute apex, base articulated to a conduplicate leaf sheath that covers the stem; the basal leaves smaller than the upper leaves. ***Inflorescence*** an apical and lateral raceme; scape 5–7 × 0.2 cm, triquetrous, up to 5 flowers, one to two flowers opening in succession. ***Floral bract*** 15–16 × 5–6 mm (progressively decreasing in size in the following bracts), conduplicate, decurrent, falcate in natural position, widely ovate when expanded, acute, carinate. ***Ovary*** 20–21 × 3.0–3.1 mm, triquetrous, winged, pedicellate. Pedicel 6–11 mm long, triquetrous. ***Flowers*** 20–25 mm in diameter, non-resupinate. ***Sepals*** greenish-yellow with red vinaceous, spots, semitranslucent; lateral sepals 11–12 × 5–6 mm, ovate, entire, basally concave, acute apex, curved in natural position, with entire margin, 3-veined, carinate abaxially; dorsal sepal 13–14 × 5–6 mm, narrowly ovate, entire, concave towards the base, acute apex, 3-veined, carinate abaxially. ***Petals*** 15–17 × 11–12 mm, red-vinaceous with spots, sub-rhombic to obovate, apex acute to sub-acute, 9–10-veined. ***Labellum*** 12–13 × 16–17 mm when expanded, red-vinaceous with spots, reniform, concave, the base embracing almost the entire column, the apex rounded, protruding, 26-veined. ***Callus*** 3.2–3.6 × 5.7–6.1 mm, elevated, dark purple towards the apex, yellow towards the base, sagittate with a rounded apex, setose at the centre of the apex, fimbriate margin. ***Column*** 3.4 mm long, 2.4 mm wide, sub-rhomboid, dark yellow, ventral surface densely covered by minute conical papillae with acute apex, 3 tufts of setae; setae dark purple to white, simple, capilliform, up to 2.2 mm long. ***Clinandrium*** concave, with rounded edges, with a dorsal projection covering up to half of the anther cap. ***Stigma*** suborbicular. ***Rostellum*** erect. ***Anther cap*** 1.7 × 2.1 mm, red, cordiform, bilocular. ***Pollinarium*** 2.4 × 1.9 mm; pollinia 4 in 2 unequal pairs; outer pair obovoid, convex-flat; inner pair ellipsoid, laterally complanate; caudicle hyaline; viscidium uncinate. ***Fruit*** not seen.

**Figure 2.**
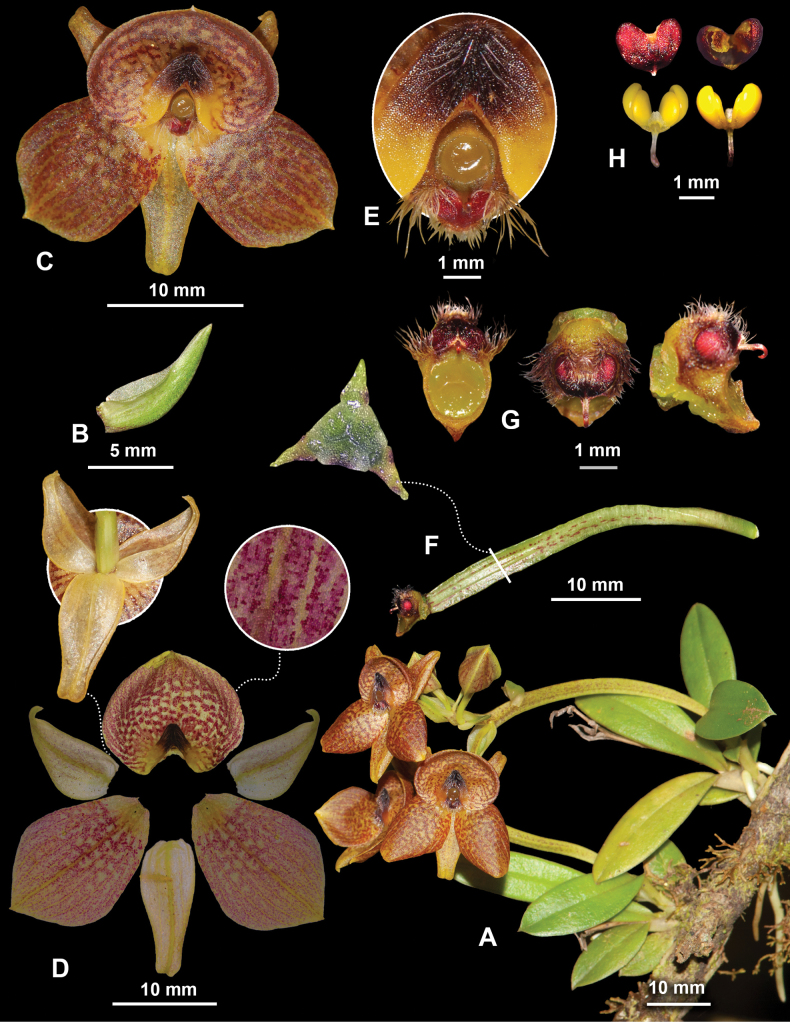
*Telipogon rojasiae* A. Habit; B. Floral bract, lateral view; C. Flower, frontal view; D. Dissected flower, frontal view with details of the abaxial side of the sepals and the labellum surface; E. Callus and column, frontal view; F. Pedicel, ovary and column, lateral view with details of the ovary cross-section; G. Column, frontal, top and lateral views; H. Anther cap and pollinarium, ventral and dorsal views. Note that all the floral segments are in their natural position, not expanded.

**Figure 3.**
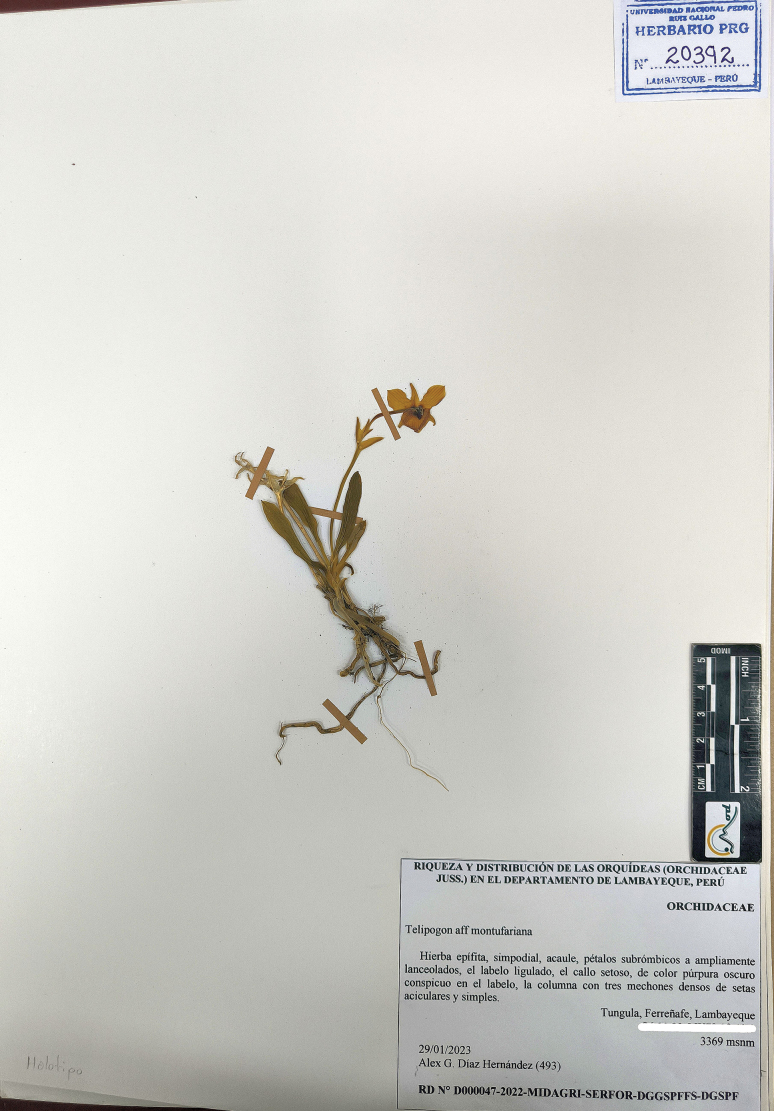
*Telipogon rojasiae*. Holotype. GPS coordinates hidden for conservation purposes.

##### Phenology.

Plants of *T. rojasiae* have been recorded in flower between August and January, the dry season in the southern hemisphere.

##### Eponymy.

*Telipogon rojasiae* is named after Dr Consuelo Rojas, professor of Botany at the Universidad Nacional Pedro Ruiz Gallo, for her work on biodiversity and plant conservation in Northern Peru.

##### Distribution, habitat and ecology.

**Figure 4.**
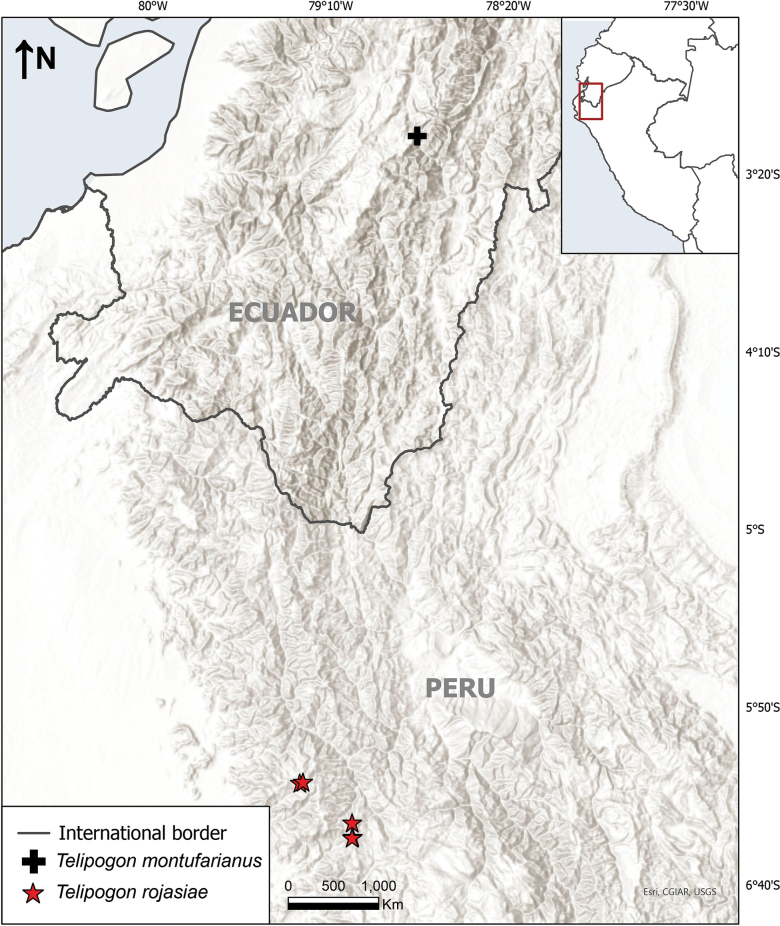
Distribution map of *Telipogon rojasiae* and the morphologically similar *T. montufarianus*.

*Telipogon rojasiae* is only known from the western slopes of the northern Peruvian Andes between 2800 and 3400 m asl (Fig. 4). The ecosystem of this area has been categorised as relict montane forests of the western slope (Ministerio del Ambiente 2019). These relict forests are located in the western Andean slopes of northwest Peru and southwest Ecuador and possess a rich biodiversity, including many endemic species and genera (Weigend et al. 2005a, 2005b).

We have so far recorded five small subpopulations, which occur in small forest patches. In these areas, *Miconia* sp. is the dominant plant species, followed by *Brachyotum ledifolium* (Desr.) Triana, *Hedyosmum scabrum* (Ruiz & Pav.) Solms, *Grosvenoria jelskii* (Hieron.) R.M.King & H.Rob., and *Myrica pubescens* Humb. & Bonpl. ex Willd. In terms of orchids, many species co-occur with *T. rojasiae*, including *Cyrtochilum loesenerianum* (Schltr.) Dalström, *Epidendrum capitellatum* C.Schweinf., *E. hemiscleria* Rchb.f., *E. scutella* Lindl., *Fernandezia ionanthera* (Rchb.f. & Warsz.) Schltr., *Liparis elegantula* Kraenzl., and *Telipogon papilio* Rchb.f. & Warsz.

The flower morphology resembles the general morphology of flowers in *Telipogon* species pollinated by sexual deception involving tachinid male flies (e.g. *T. peruvianus* T.Hashim., *T. bowmanii* Rchb.f.; Martel et al. 2016, 2019), which suggests *T. rojasiae* may have a similar pollination mechanism.

##### Preliminary conservation status.

*Telipogon rojasiae* was first observed in 2016; since then, only five small subpopulations, each comprising fewer than 20 individuals, have been recorded. The five subpopulations occur in disturbed and patchy forests, which are threatened by deforestation. Its EOO is estimated to be 132.5 km^2^, falling into the Endangered category under subcriterion B1, whereas its AOO is estimated at 5 km^2^ (a cell of 1 km^2^ due to the small forest remnants), falling into the Critically Endangered category under subcriterion B2. The known habitat of *T. rojasiae*, the relict forests, is severely fragmented (a), and we project that the five known subpopulations will decline over time and the AOO and EOO will be reduced. Therefore, following the IUCN (2024) criteria, we informally assessed the conservation status of *T. rojasiae* as Critically Endangered [CR B2ab(i,ii,iii,iv)].

##### Taxonomic notes.

**Figure 5.**
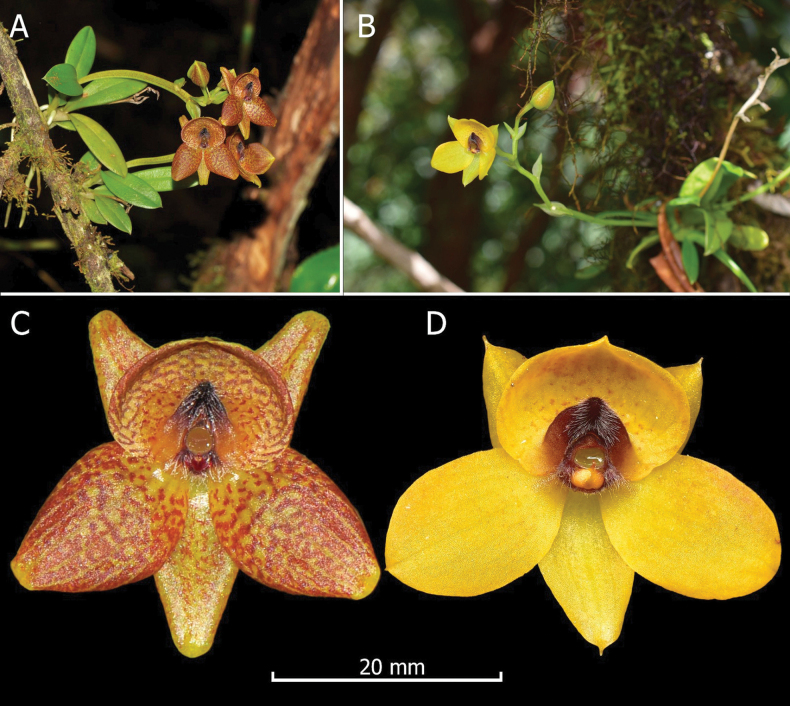
Comparison between *Telipogon rojasiae* and *T. montufarianus*; A. Plant of *T. rojasiae*; B. Plant of *T. montufarianus*; C. Flower of *T. rojasiae*, frontal view; D. Flower of *T. montufarianus*, frontal view.

*Telipogon rojasiae* is morphologically most similar to *T. montufarianus* H.Medina, J.Portilla & Hirtz, for being a medium-sized plant (< 10–15 cm including the inflorescence) and having small flowers of similar size (20–25 mm in diam.), a similar colouration in the sepals, the concave labellum, an elevated callus, and a column with three tufts of capilliform setae (Fig. 5). Some of the differences between both species have been detailed in the diagnosis; however, the most notable are the colour of the flowers (cream-yellow flowers heavily stained with red vinaceous in *T. rojasiae* vs. bright yellow flowers in *T. montufarianus*), the shape of the petals (sub-rhombic to obovate petals vs. elliptic), the shape of the callus (sagittate in *T. rojasiae* vs. widely sub-cordiform in *T. montufarianus*), the number of veins in the petals (10 veins in *T. rojasiae* vs. 5 veins in *T. montufarianus*) and the labellum (26 veins in *T. rojasiae* vs. 16–19 veins in *T. montufarianus*) (Fig. 4). Additionally, *T. rojasiae* inhabits the montane forests of the eastern Andean slopes of northwestern Peru, while *T. montufarianus* is distributed in the western slopes of southeastern Ecuador (Table 1). *Telipogon rojasiae* seems to be also morphologically similar to *Telipogon frymirei* Dodson, from southeastern Ecuador, as they both have a concave labellum and an elevated callus; however, *T. rojasiae* can be easily discriminated by the sub-rhombic to sub-obovate petals (vs. elliptic petals in *T. frymirei*), the reniform labellum (vs. broadly ovate in *T. frymirei*), the sagittate callus (vs. sub-triangular, lingulate callus in *T. frymirei*), and the rounded margins of the stigmatic cavity (vs. a stigmatic cavity with incurved teeth in *T. frymirei*) (Table 1).

**Table 1.** Summary of principal differences between *T. rojasiae* and morphologically similar species.

	T. rojasiae	T. montufarianus	T. frymirei
Flower colour	cream-yellow with red vinaceous stains	bright yellow with brown striations	yellow-brown with brown veins
Petals	sub-rhombic to obovate, 10-veined	elliptic, 5-veined	elliptic, 7-veined
Labellum	reniform, 26-veined	reniform, 16-19-veined	broadly ovate, subcordate at the base, 19-23-veined
Callus	sagittate, hirsute on the sides, setose apically	widely sub-cordiform, hirsute on the sides, setose apically	sub-triangular, lingulate, puberulent
Column	sides of the stigmatic cavity smooth	sides of the stigmatic cavity smooth	borders of the stigmatic cavity with incurved teeth

There are at least two *Telipogon* species that might be closely related to *T. rojasiae*: *T. montufarianus* and an undescribed *Telipogon* species presented as *T. frymirei* Dodson by Dodson (2002) in his book Native Ecuadorian Orchids, volume III. The latter displays the typical concave labellum and a hairy elevated callus, as *T. rojasiae* and *T. montufarianus* do; in addition, it has dark red to purplish flowers. These and other characters of the specimen portrayed by Dodson (2002) differ strongly from the characters presented in the original description and drawing of *T. frymirei* (Dodson 1984). The misidentified species resembles *T. montufarianus* in petal shape and *T. rojasiae* in labellum shape. Unfortunately, no material is available to compare our new entity with the actual and misidentified *T. frymirei*. Moreover, in the same book, Dodson depicted a picture of a flower of *T. montufarianus* – which was still undescribed at that time – as *T. ecuadorensis* Schltr., despite these two species being morphologically very distinct. Explorations in the type locality of *T. frymirei* are being carried out, which we hope will shed light on the true identity of this and other *Telipogon* species, as well as the potential occurrence of *T. rojasiae* in Ecuador.

As a side note, *T. montufarianus* was originally described as *T. montufariana* (Medina et al. 2019), which is an incorrect declension, since the genus name *Telipogon* is masculine, and therefore the species epithet should be declined with the suffix “ianus”. Similarly, *T. humboldtianus* H.Medina, J.Portilla & Hirtz was originally named *T. humboldtiana* (Medina et al. 2019), which we correct here.

##### Other records.

Peru • Cajamarca. Prov. Chota, [Dist. Querocoto,] Querocoto, 3144 m, 20 Aug 2017, *Alex G. Diaz H. 271* (PRG! [Accession N° 20391]); • ibid. 3148 m, 20 Sep 2018, *Alex G. Diaz H. 270* (PRG! [Accession N° 20390]); • ibid. Pagaibamba, 2808 m, 11 Aug 2018, *Alex G. Diaz H. 268* (PRG! [Accession N° 20389]); • ibid. Lambayeque. Prov. Ferreñafe, [Dist. Incahuasi,] Tungula, 3400 m, 17 Oct 2016, *Alex G. Diaz H. 128* (PRG! [Accession N° 18754]).

## Supplementary Material

XML Treatment for Telipogon rojasiae

## ﻿Additional information

### Use of AI

No use of AI was reported.

### Author ORCIDs

Carlos Martel https://orcid.org/0000-0001-9892-1999

Alex G. Diaz Hernández https://orcid.org/0000-0002-3892-6154

Gabriel A. Iturralde https://orcid.org/0000-0003-2456-0929

Benjamín Collantes https://orcid.org/0000-0003-2346-9576

### Data availability

All of the data that support the findings of this study are available in the main text.

## References

[B1] Bachman S Moat J Hill A de la Torre J Scott B (2011) Supporting Red List threat assessments with GeoCAT: Geospatial conservation assessment tool. ZooKeys 150: 117–126. 10.3897/zookeys.150.2109 PMC3234434 22207809

[B2] Beentje H (2012) The Kew Plant Glossary: An Illustrated Dictionary of Plant Terms. Royal Botanic Gardens, Kew. Kew Publishing, Richmond, UK, 160 pp.

[B3] Chamaya Gonzáles JA (2023) Diversidad de especies de la familia Orchidaceae en el bosque La Palma – Chota – Cajamarca. MSc thesis. Universidad Nacional de Cajamarca, Peru.

[B4] Dodson CH (1984) *Telipogon frymirei*. In: Dodson CH, Dodson PM (Eds) Icones Plantarum Tropicarum, Fascicle 10. The Marie Selby Botanical Gardens, Sarasota, Plate 991.

[B5] Dodson CH (2002) Native Ecuadorian Orchids. Volume III: *Lepanthopsis*-*Oliveriana*. Dodson Trust, Sarasota, 231 pp.

[B6] Iturralde GA Monteros MF Jiménez MM Martel C Baquero LE (2023) *Telipogon pillaropatatensis* (Oncidiinae): A new species from the east-central Andes of Ecuador. Lankesteriana 23: 495–509. 10.15517/lank.v23i3.58100

[B7] IUCN (2024) IUCN Standards and Petitions Committee 2024. Guidelines for Using the IUCN Red List Categories and Criteria. Version 16. Prepared by the Standards and Petitions Committee. https://www.iucnredlist.org/resources/redlistguidelines.pdf [Accessed 01 July 2025]

[B8] Jiménez MM Martel C Garzón-Suárez HX Baquero LE Mashendo V Iturralde GA (2024) *Telipogon leisberthvelezii* (Orchidaceae: Oncidiinae), a new orchid species from the Cordillera del Cóndor in Ecuador. Kew Bulletin 79: 97–106. 10.1007/s12225-023-10145-5

[B9] Martel C (2016) New records for two Peruvian endemic *Telipogon* (Orchidaceae) including an unexpected record of *Telipogon ariasii*. Revista Peruana de Biología 23: 43–36. 10.15381/rpb.v23i1.11832

[B10] Martel C Nauray Huari W (2013) Notes and emended description of *Telipogon peruvianus* T. Hashim. (Orchidaceae). Candollea 68: 245–250. 10.15553/c2012v682a8

[B11] Martel C Cairampoma L Stauffer FW Ayasse M (2016) *Telipogon peruvianus* (Orchidaceae) flowers elicit pre-mating behaviour in *Eudejeania* (Tachinidae) males for pollination. PLOS ONE 11: e0165896. 10.1371/journal.pone.0165896 PMC5094723 27812201

[B12] Martel C Francke W Ayasse M (2019) The chemical and visual bases of the pollination of the Neotropical sexually deceptive orchid *Telipogon peruvianus*. New Phytologist 223: 1989–2001. 10.1111/nph.15902 31074029

[B13] Martel C Edquen J Collantes B Ocupa L (2020) *Telipogon chachapoyensis* (Orchidaceae), a new species from Peru similar to *T. microglossus*. Systematic Botany 45: 227–232. 10.1600/036364420X15862837791285

[B14] Medina H Portilla J Hirtz A (2019) New Ecuadorian orchids – Two new species in Oncidiinae and a new species in Zygopetalinae. Lindleyana 88: 546–552.

[B15] Ministerio del Ambiente (2019) Mapa Nacional de Ecosistemas del Perú: Memoria Descriptiva. Ministerio del Ambiente, Lima, 124 pp.

[B16] Nauray HW Galán A (2008) Ten new species of *Telipogon* (Orchidaceae, Oncidiinae) from southern Peru. Anales del Jardín Botánico de Madrid 65: 73–95. 10.3989/ajbm.2008.v65.i1.247

[B17] POWO (2025) Plants of the World Online. Facilitated by the Royal Botanic Gardens, Kew. http://www.plantsoftheworldonline.org [Accessed 15 June 2025]

[B18] Pupulin F (2022) Vanishing Beauty: Native Costa Rica Orchids. Vol. 3. *Restrepia*-*Zootrophion* and appendices. Koeltz Botanical Books, Oberreifenberg, 467 pp.

[B19] Weigend M Cano A Rodríguez EF (2005a) New species and new records of the flora in Amotape-Huancabamba Zone: Endemics and biogeographic limits. Revista Peruana de Biología 12: 249–274. 10.15381/rpb.v12i2.2398

[B20] Weigend M Rodríguez EF Arana C (2005b) The relict forests of Northwest Peru and Southwest Ecuador. Revista Peruana de Biología 12: 185–194. 10.15381/rpb.v12i2.2390

